# Whole-Genome Assessment of Clinical *Acinetobacter baumannii* Isolates Uncovers Potentially Novel Factors Influencing Carbapenem Resistance

**DOI:** 10.3389/fmicb.2021.714284

**Published:** 2021-10-01

**Authors:** Kiran Javkar, Hugh Rand, Maria Hoffmann, Yan Luo, Saul Sarria, Nagarajan Thirunavukkarasu, Christine A. Pillai, Patrick McGann, J. Kristie Johnson, Errol Strain, Mihai Pop

**Affiliations:** ^1^Department of Computer Science, University of Maryland, College Park, MD, United States; ^2^Joint Institute for Food Safety and Applied Nutrition, University of Maryland, College Park, MD, United States; ^3^Center for Food Safety and Applied Nutrition, United States Food and Drug Administration, Department of Health and Human Services, College Park, MD, United States; ^4^Center for Veterinary Medicine, United States Food and Drug Administration, Department of Health and Human Services, Laurel, MD, United States; ^5^Multidrug Resistant Organism Repository and Surveillance Network, Walter Reed Army Institute of Research, Silver Spring, MD, United States; ^6^Department of Pathology, University of Maryland School of Medicine, Baltimore, MD, United States

**Keywords:** whole-genome comparison, antibiotic resistance, genomic association studies, multiple genome alignment, *Acinetobacter baumanni*

## Abstract

Carbapenems—one of the important last-line antibiotics for the treatment of gram-negative infections—are becoming ineffective for treating *Acinetobacter baumannii* infections. Studies have identified multiple genes (and mechanisms) responsible for carbapenem resistance. In some *A. baumannii* strains, the presence/absence of putative resistance genes is not consistent with their resistance phenotype—indicating the genomic factors underlying carbapenem resistance in *A. baumannii* are not fully understood. Here, we describe a large-scale whole-genome genotype-phenotype association study with 349 *A. baumannii* isolates that extends beyond the presence/absence of individual antimicrobial resistance genes and includes the genomic positions and pairwise interactions of genes. Ten known resistance genes exhibited statistically significant associations with resistance to imipenem, a type of carbapenem: *blaOXA-23, qacEdelta1, sul1, mphE, msrE, ant(3”)-II, aacC1, yafP, aphA6*, and *xerD*. A review of the strains without any of these 10 genes uncovered a clade of isolates with diverse imipenem resistance phenotypes. Finer resolution evaluation of this clade revealed the presence of a 38.6 kbp conserved chromosomal region found exclusively in imipenem-susceptible isolates. This region appears to host several HTH-type DNA binding transcriptional regulators and transporter genes. Imipenem-susceptible isolates from this clade also carried two mutually exclusive plasmids that contain genes previously known to be specific to imipenem-susceptible isolates. Our analysis demonstrates the utility of using whole genomes for genotype-phenotype correlations in the context of antibiotic resistance and provides several new hypotheses for future research.

## 1. Introduction

Antimicrobial resistance (AMR) is a grave healthcare challenge worldwide, causing 700,000 deaths each year (Review on Antimicrobial Resistance, 2016). A sizable proportion of AMR bacterial infections are hospital-acquired and pose a high healthcare burden in developed and developing countries alike (Chen et al., 2015). Some of these bacterial infections can be resistant to even the most powerful antibiotics available; including carbapenems which are typically considered to be one of the “last-line” antibiotics by clinicians, and specifically used in the treatment of critically-ill patients affected potentially by antimicrobial-resistant Gram-negative infections (Codjoe and Donkor, 2017). Antimicrobial resistance is caused by a wide variety of genetic factors across different pathogens; discovering and understanding these factors has been accelerated by the advancements in whole genome sequencing technologies and the development of methods for analyzing the resulting data (Boolchandani et al., 2019).

*Acinetobacter baumannii*, a Gram-negative and frequently multidrug-resistant pathogen, is an important nosocomial pathogen worldwide (Hamidian and Nigro, 2019). The Infectious Diseases Society of America considers *A. baumannii* to be among the top 6 leading causes of nosocomial infections, i.e., one of the high-priority ESKAPE pathogens (Boucher et al., 2009). The World Health Organization has marked carbapenem-resistant *Acinetobacter baumannii* (CRAB) as their highest priority pathogen for the research and development of novel antibiotics (Tacconelli et al., 2018). *A. baumannii* has the ability to proliferate readily in hospitals, particularly in intensive care units, and spread epidemically among patients (Chen et al., 2015). It can cause a broad range of severe infections, such as skin and soft tissue infections, wound infections, urinary tract infections, secondary meningitis, and ventilator-associated pneumonia (Roca et al., 2012; Nowak et al., 2017). Its ability to have intrinsic resistance and propensity to acquire resistance has led to the emergence of multidrug-resistant, extensively drug-resistant, and pan-drug resistant *A. baumannii* strains (Peleg et al., 2008; Evans et al., 2013). An increasing rate of carbapenem resistance was documented among *A. baumannii* strains from numerous hospital outbreaks (Zarrilli et al., 2013; Ben-Chetrit et al., 2018).

*A. baumannii* strains have the ability to resist the action of carbapenems via both intrinsic properties and acquired resistance factors (Evans et al., 2013). Carbapenem resistance is primarily mediated by the production of carbapenem-hydrolyzing beta-lactamases, also called carbapenemases (Poirel and Nordmann, 2006; Roca et al., 2012; Pagano et al., 2016). Beta-lactamases have been historically categorized into four molecular classes (A to D) based on conserved amino-acid motifs. Carbapenem resistance in *A. baumannii* is largely attributed to the class D beta-lactamases, also known as OXA-type carbapenemases (Evans and Amyes, 2014). The other major mechanism conferring carbapenem resistance in *A. baumannii* is the modification of membrane permeability, either by the loss or decrease in the expression of outer membrane proteins, increased expression of efflux pumps, or modifications in penicillin-binding proteins (PBPs) (Kröger et al., 2016). The known factors that influence carbapenem resistance in *A. baumannii* are described in Table 1.

**Table 1:** Genomic drivers known to influence carbapenem resistance in *Acinetobacter baumannii*.

**Genetic element**	**Mechanism for** **carbapenem** **resistance**	**Literature** **supporting** **gene-resistance** **Link**	**Literature** **indicating** **insufficient** **support**
Carbapenem hydrolysis by beta-lactamase genes			
Intrinsic beta-lactamases (*OXA-51-like, ADC*, etc.)	Weak hydrolysis Primarily chromosomal	Corvec et al., 2003 Turton et al., 2006 Schroder et al., 2016	Antunes et al., 2014 Héritier et al., 2005a Takebayashi et al., 2020
Acquired beta-lactamases (*OXA-23, OXA-58*, etc.)	Stronger hydrolysis Primarily plasmid-encoded	Antunes et al., 2014 Pagano et al., 2016 Héritier et al., 2005b	Carvalho et al., 2011 Boo and Crowley, 2009 Wallace et al., 2016
Insertion Sequences (IS*Aba1*, IS*Aba3*, etc.)	Upregulates the expression of beta-lactamase genes when present in their promoter region	Evans and Amyes, 2014 Cayô et al., 2011 Takebayashi et al., 2020	Khorsi et al., 2015 Pagano et al., 2013
Loss of membrane permeability			
Outer membrane proteins (*OmpA, CarO*)	Mediated by the loss or inactivation of genes	Mussi et al., 2005 Jeong et al., 2009	Zhao et al., 2015
Penicillin-binding proteins (PBPs)	Mediated by the modification or loss of PBPs	Vashist et al., 2011 Hawkey et al., 2018	Cayô et al., 2011
Efflux pumps (*AdeABC, AdeIJK*, etc.)	Overexpression of efflux pumps	Jia et al., 2015 Héritier et al., 2005b	Chang et al., 2014 Liu et al., 2014

*Citations are provided that both support the link of individual genes with resistance and that suggest that specific genes are not sufficient to confer resistance*.

CRAB strains are also resistant to a number of non-carbapenem antibiotics. Mechanisms of resistance include aminoglycoside-modifying enzymes, sulfonamide resistant genes, quaternary amines, other resistance genes encoded within the AbaR resistance islands (Taitt et al., 2014; Pagano et al., 2016). Similarly, CRAB strains may encode transcriptional regulator and transporter genes which are known to expedite resistance to non-carbapenem antibiotics by regulating the expressions of influx/efflux pumps, biofilm formation, outer membrane permeability, etc. (Gordon and Wareham, 2010; Chang et al., 2014) The impact of these elements on carbapenem resistance has not been fully quantified, and they do occur in carbapenem-susceptible isolates as well (Bratu et al., 2008; Bi et al., 2019). Overall, the resistance phenotype of an isolate is likely a product of the interactions of different genomic factors, and we currently lack a full understanding of the complete set of such genomic factors and their interactions. Here, we utilize a large-scale whole-genome comparison to address some gaps in our understanding of carbapenem resistance in *A. baumannii*.

Our work expands upon the findings of a recent study of 203 *A. baumannii* isolates collected as a part of an active infection control surveillance program at the University of Maryland Medical Center (UMMC) (Wallace et al., 2016). We increase the number of genomes analyzed to 349 (Supplementary Table 1 for NCBI Accessions) and expand the analysis beyond individual genes and conventional genome-wide association studies (GWAS). The analysis of the association between genotype and phenotype encompasses known resistance genes, insertion sequences, plasmids, single nucleotide polymorphisms (SNPs), and other conserved regions. Of particular interest is a subset of genetically similar strains for which the resistance phenotype could not be explained by the known determinants of carbapenem resistance. In this subset, we employ sophisticated whole genome analysis approaches and identify several genomic features that are associated with antibiotic resistance within these isolates—providing several intriguing opportunities for future research.

## 2. Results

### 2.1. Genomic Data, Antimicrobial Susceptibility Testing, and MLST

The 349 *A. baumannii* isolates were collected in clinical setting as a part of a surveillance program at the University of Maryland Medical Center (UMMC); these isolates had been sequenced and are accessible through NCBI (Supplementary Table 1). The level of resistance to several carbapenems was obtained using the Kirby-Bauer disk diffusion method, and phenotypes were determined according to the Clinical Laboratory Standards Institute (CLSI) breakpoints (CDC, 2017). Here, we restrict our analysis to imipenem, for which information was available for all isolates (Supplementary Table 1). Out of the 349 isolates, 305 were imipenem-resistant and 44 were imipenem-susceptible. Since the sequence assemblies available at NCBI were highly fragmented (mean contig count: 145) and had a higher number of misassemblies (mean misassemblies count: 84, mean mismatches per 100 kbp count: 1,003), we re-assembled the sequencing reads for each isolate (mean contig count: 126, mean misassemblies count: 59, mean mismatches per 100 kbp count: 729) and re-annotated the resulting assemblies, as described in Methods.

MLST sequence types (ST) were determined for the isolates using the Pasteur scheme (Diancourt et al., 2010); the resultant MLST profiles are presented in Supplementary Table 1. Two hundred and thirty-six out of the three hundred and forty-six isolates were assigned to the Pasteur ST 2, which was also the largest ST category identified in Wallace et al. (2016) study.

### 2.2. The Presence of Known Antimicrobial Resistance Genes

Known antimicrobial resistance (AMR) genes were found in all isolates (Supplementary Table 2). Some of the known AMR genes exhibited a strong association with the imipenem-resistant phenotype (*p* ≤ 0.01, Fisher’s exact test), but were also found in many imipenem-susceptible isolates as well (Supplementary Table 2), indicating that alone those genes do not explain resistance. To identify genes that are most likely to confer resistance in the absence of other factors, we focused on genes with strong statistical association with the imipenem-resistance phenotype (*p* ≤ 0.01) and which were infrequently found in imipenem-susceptible isolates (≤ 10 out of 44). Ten AMR genes were strongly correlated with the imipenem-resistant phenotype according to these criteria (Table 2 and Supplementary Table 1). One or more of these genes was identified in 294 (84%) *A. baumannii* isolates (288 imipenem-resistant and 6 imipenem-susceptible), suggesting that for the majority of the isolates (82%) the imipenem-resistant phenotype can be explained by their presence.

**Table 2:** Ten known antimicrobial resistance (AMR) genes strongly associated with imipenem-resistance in 349 *A. baumannii* genomes analyzed (*p* ≤ 0.01, Fisher’s exact test) and present infrequently in imipenem-susceptible isolates (≤10 out of 44).

**Gene** **annotation**	**Gene present**		**Gene absent**		**Odds** **ratio**
	**Resistant**	**Susceptible**	**Resistant**	**Susceptible**	
*blaOXA-23*	182 (60%)	1 (2%)	123 (40%)	43 (98%)	63.62
*qacEdelta1*	257 (84%)	4 (9%)	48 (16%)	40 (91%)	53.54
*sul1*	259 (85%)	5 (11%)	46 (15%)	39 (89%)	43.91
*mphE*	175 (57%)	2 (5%)	130 (43%)	42 (95%)	28.27
*msrE*	174 (57%)	2 (5%)	131 (43%)	42 (95%)	27.89
*ant(3”)-II*	158 (52%)	2 (5%)	147 (48%)	42 (95%)	22.57
*aacC1*	156 (51%)	1 (2%)	149 (49%)	43 (98%)	45.02
*yafP*	155 (51%)	1 (2%)	150 (49%)	43 (98%)	44.43
*aphA6*	74 (24%)	1 (2%)	231 (76%)	43 (98%)	13.77
*xerD*	72 (24%)	1 (2%)	233 (76%)	43 (98%)	13.29
Combined total	288 (94%)	6 (14%)	17 (6%)	38 (86%)	107.29

Wallace et al.’s analysis of 203 *A. baumannii* isolates identified eight genes not previously considered to be AMR genes that were strongly associated with carbapenem resistance (Wallace et al., 2016). Within the larger set of 349 isolates (that includes the 203 strains analyzed by Wallace et al.), these genes continued to be significantly-associated with resistance (Table 3). Seven of these eight genes were detected exclusively in imipenem-resistant isolates—each of these isolates also contained one or more of the 10 strongly correlated AMR genes (Table 2).

**Table 3:** Prevalence within our collection of isolates of genes found by Wallace et al. (2016) to be associated with imipenem-resistance in *A. baumannii*.

**Gene ID**	**Annotation**	**Gene present**		**Gene absent**	
		**R**	**S**	**R**	**S**
55125	Polysaccharide biosynthesis family protein	101 (33%)	0 (0%)	204 (67%)	44 (100%)
74448	Glycosyl transferases group 1 family protein	102 (33%)	0 (0%)	203 (67%)	44 (100%)
197234	KR domain protein	102 (33%)	0 (0%)	203 (67%)	44 (100%)
197235	Cytidylyltransferase family protein	102 (33%)	0 (0%)	203 (67%)	44 (100%)
365901	Type IV pilin structural subunit	51 (17%)	18 (41%)	254 (83%)	26 (59%)
563378	Conserved hypothetical protein	102 (33%)	0 (0%)	203 (67%)	44 (100%)
733161	Oxidoreductase, NAD-binding Rossmann fold family protein	101 (33%)	0 (0%)	204 (67%)	44 (100%)
733163	Capsule polysaccharide biosynthesis family protein	101 (33%)	0 (0%)	204 (67%)	44 (100%)

*(R) and (S) denote the imipenem-resistant and imipenem-susceptible isolates, respectively. Gene IDs correspond to the identifiers assigned to the genes in the original study. Seven out of these eight genes (excluding the gene with gene id: 365901) were present in isolates that also contained one or more of the 10 strongly correlated AMR genes from Table 2*.

### 2.3. The Impact of Insertion Sequence IS*Aba1* on Resistance

Two beta-lactamase genes known to elevate resistance to carbapenems in *A. baumannii, blaOXA-65-like* (*blaOXA-51* family) and *blaADC*, were found in all 349 isolates analyzed in our study, indicating their presence alone is not sufficient for resistance. These two genes in conjunction with an upstream insertion of a mobile genetic element, the IS*Aba1* insertion sequence, have been previously associated with resistance (Table 1). However, the analysis of insertion sequences and other mobile elements is complicated due to the challenges they pose to assembly algorithms (Ashton et al., 2015; Adams et al., 2016), a fact recapitulated in our data where IS*Aba1* was identified predominantly on a separate genomic contig. To provide a genomic context for this sequence with respect to other resistance genes, we relied on scaffolding information based on paired-reads. This information allowed us to ascertain the relative distance between IS*Aba1* and resistance genes (see Supplementary Table 3). The IS*Aba1* sequence was detected in 317 of the 349 isolates, where 296 are resistant and 21 are susceptible. As shown in Table 4, the presence of IS*Aba1* in the upstream region of the *blaOXA-65-like* and *blaADC* genes was strongly associated with resistance (*p* ≤ 0.01, Fisher’s exact test), and we also recapitulated the strong association between IS*Aba1* and *blaOXA-23*.

**Table 4:** Imipenem resistance is potentiated by the presence of the IS*Aba1* insertion sequence upstream of beta-lactamase genes.

**Gene** **annotation**	**Both, IS*****Aba1*** **and gene, present**				**IS*****Aba1*** **absent**, **gene present**	
	**IS*****Aba1*** **is upstream** **of the gene**		**IS*****Aba1*** **is not** **upstream of the gene**			
	**Resistant**	**Susceptible**	**Resistant**	**Susceptible**	**Resistant**	**Susceptible**
*blaOXA-23*	110 (36%)	0 (0%)	72 (24%)	1 (2%)	0 (0%)	0 (0%)
*blaOXA-65*	148 (49%)	0 (0%)	148 (49%)	21 (48%)	9 (2%)	23 (52%)
*blaADC*	145 (48%)	2 (5%)	151 (50%)	19 (43%)	9 (2%)	23 (52%)

*The percentages denote the fraction of the isolates with the imipenem-resistance phenotype within the respective categories from the total number of isolates with the corresponding phenotype (305 imipenem-resistant and 44 imipenem-susceptible)*.

### 2.4. Microbial Clade Lacking Known Antimicrobial Resistance Genes

**Figure 1:**
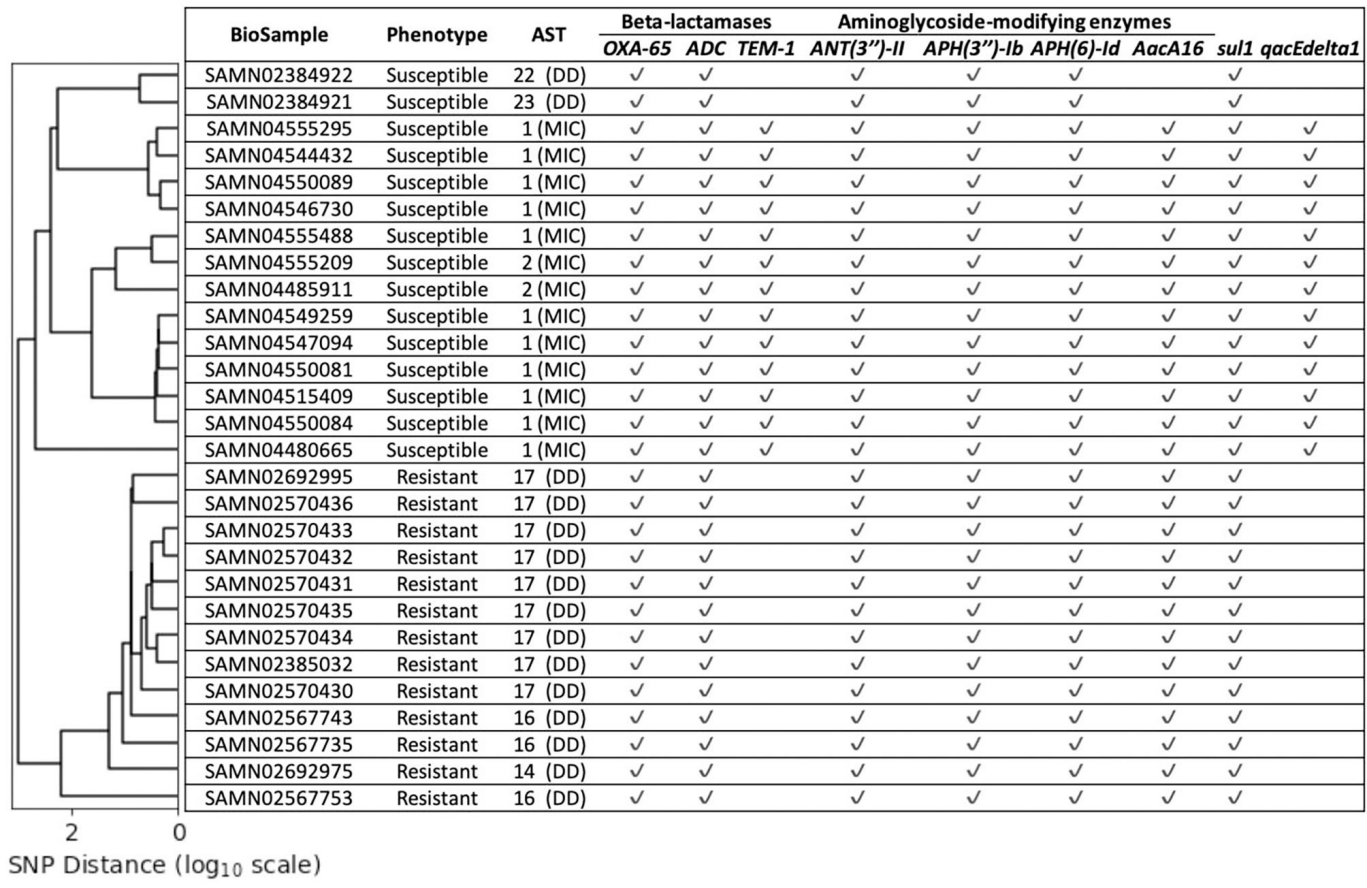
The lineage estimated for the chosen 28 isolates (y-axis) based on SNP distance [x-axis (log10 scale)]. The Antimicrobial Susceptibility Testing (AST) values provide the imipenem susceptibilities for the corresponding isolates measured via disk diffusion (DD) or minimum inhibitory concentration (MIC). None of these isolates were predicted to contain any of the 10 strongly correlated known AMR genes presented in Table 2, but contain several other known AMR genes. The imipenem-resistant and susceptible isolates cluster separately.

Fifty five isolates (17 imipenem-resistant and 38 imipenem-susceptible) did not encode either of the 10 genes we have found to be strongly associated with imipenem-resistance. The imipenem resistance within the resistant isolates could not be directly explained by the known AMR genes, or the presence of the IS*Aba1* insertion sequence in the upstream region of the beta-lactamase genes. We estimated the phylogeny of all 55 isolates based on the single nucleotide polymorphism (SNP) matrix computed with the reference-based CFSAN SNP pipeline (Supplementary Figure 1) (Davis et al., 2015). This analysis revealed a clade that comprised 15 isolates: 13 of the 17 imipenem-resistant isolates and 2 imipenem-susceptible isolates. All isolates from this clade displayed similar known AMR gene profiles (Figure 1). In order to better explore the genomic differences between the imipenem-resistant and susceptible isolates from this clade, an additional 13 sequences of imipenem-susceptible strains were retrieved from the NCBI Pathogen Detection Isolates Browser (NCBI, 2021). These *A. baumannii* isolate sequences were located in the same SNP cluster as the genome sequences from the above-mentioned clade (cluster ID: PDS000005681). The NCBI identifiers for these 13 isolates along with their carbapenem susceptibilities and phenotypes are provided in Supplementary Table 4. As we have done for all isolates discussed in this paper, their sequences were re-assembled and subjected to gene prediction and annotation. Furthermore, we were able to obtain biological samples from 12 of these isolates and confirm the phenotype reported in the NCBI database (susceptible to imipenem, Supplementary Table 4).

To assess whether known determinants of resistance, beyond the 10 genes described above, could determine the phenotype of these 28 isolates (15 from our study plus 13 retrieved from NCBI), we predicted their phenotype using PATRIC (v3.6.5 online) (Wattam et al., 2017). The computational predictions disagreed with the experimentally-determined phenotypes: all 13 imipenem-resistant isolates were predicted by PATRIC to be susceptible, while 3 of the imipenem-susceptible isolates were predicted to be resistant (Supplementary Table 5).

### 2.5. Pan-Genome Analysis for Genomic Variants Associated With Resistance Phenotypes

The 28 isolates had high overlap in gene content—among the 4,092 genes within their collective pan-genome, 3,316 (81%) constituted the core genes (genes found in at least 99% of these genomes). Among the core genes were several known AMR genes and virulence factors (Supplementary Table 6). All 28 isolates were assigned to the Pasteur ST 2 MLST profile. Despite the high genomic similarity between these isolates, the imipenem-resistant isolates clustered within a clade distinct from that of the imipenem-susceptible ones (Figure 1). The insertion sequence IS*Aba1* was detected upstream of the beta-lactamase genes *blaOXA-65-like* as well as *blaADC* in imipenem-resistant as well as imipenem-susceptible isolates (Supplementary Table 6). The Antimicrobial Susceptibility Testing (AST) values for these 28 isolates (Figure 1) were extremely close to the CLSI breakpoints that determined the respective imipenem-resistance phenotypes-suggesting the presence of genomic variants that influence the imipenem susceptibilities just enough to alter the phenotype in the absence of strongly associated imipenem-resistance genes.

**Figure 2:**
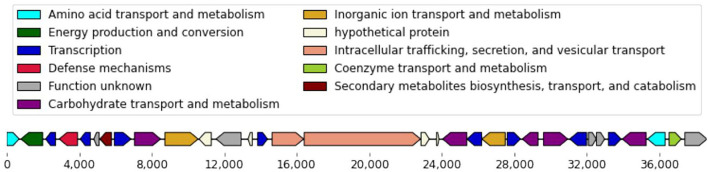
Genes encoded in the 38,651 nt chromosomal gene cassette. Within the chosen 28 isolates, this chromosomal gene cassette was conserved in all 15 imipenem-susceptible isolates and absent from all 13 imipenem-resistant isolates.

We performed a whole-genome comparison of the isolates to identify genomic regions that are present exclusively in resistant or susceptible isolates. To improve our chances of detecting contiguous genomic segments that may contribute to imipenem-resistance, we re-sequenced 6 of the isolates using the long read Pacific Biosciences technology, obtaining complete genome sequences for these isolates (Supplementary Table 4). This analysis revealed a group of transcriptional regulators and transporters that were found exclusively within the imipenem-susceptible isolates. The transcriptional regulators were predominantly helix-turn-helix (HTH) type DNA binding transcriptional regulators, such as *GntR* family, *IclR* family, *LysR* family, *MarR* family, and *TetR* family. These genes were encoded in a 38,651 nt contiguous chromosomal region (Figure 2)—this region is conserved across all 15 imipenem-susceptible isolates and absent from all 13 imipenem-resistant isolates (Supplementary Table 5).

Whole-genome resequencing of the isolates also allowed us to identify two plasmids with mutually-exclusive presence (Supplementary Table 5). The longer plasmid is 111 kbp in length and is nearly identical to the *Acinetobacter baumannii* plasmid *pAYP-A2* (Hawkey et al., 2018) (100% query coverage, 99.99% identity). This plasmid sequence was identified in 14 of the 15 imipenem-susceptible isolates and was missing from the remaining 14 isolates. This plasmid contained a number of genes identified by Wallace et al. (2016) to be associated with susceptibility to imipenem (Supplementary Table 2). All 14 isolates that lacked this plasmid contained another plasmid of length 14.6 kbp length. The shorter plasmid was most similar to an *A. baumannii* plasmid *pORAB01-3* with 75% query coverage and 99.9% identity (Hujer et al., 2017). Neither plasmid encodes any of the known AMR genes. Note that the previously sequenced *pORAB01-3* plasmid contains an *OXA-134* family class D beta-lactamase gene *OXA-237* flanked on either side by IS*Aba1* elements, feature absent from the corresponding plasmid identified in our isolates.

**Figure 3:**
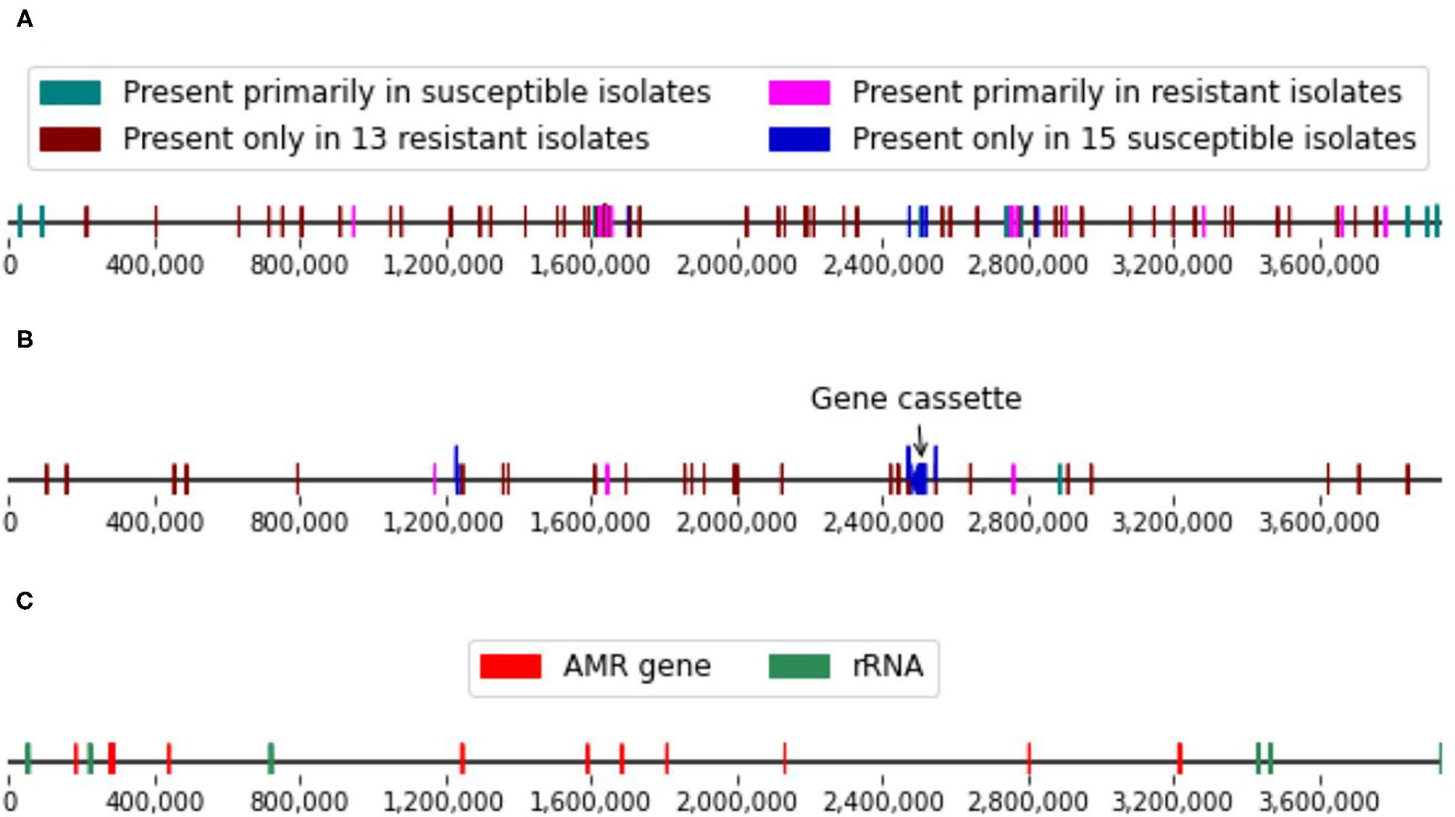
**(A)** Single nucleotide polymorphism (SNP) and **(B)** conserved genomic regions (≥ 500 nt in length) strongly associated with the imipenem-resistance phenotypes and located on the reference genome. **(C)** Locations of the known resistance genes and ribosomal RNA genes in the reference genome.

In addition to the 38.6 kbp chromosomal gene cassette and the two plasmids, the whole-genome comparisons also allowed us to locate a number of genomic variants that were strongly associated with the imipenem-resistant or susceptible isolates (*p* ≤ 0.01, Fisher’s exact test). These genomic variants included both SNPs and longer genomic segments of up to thousands of nucleotides in length. These include 75 distinct conserved regions that were at least 500 nt in length. Fourteen of these regions were also found in the reference *A. baumannii* strain [Genbank ID: NC_017162] along with 254 SNPs (Supplementary Table 8). These features were distributed fairly evenly across the reference genome (Figure 3).

## 3. Discussion

Previous studies on the association of genetic elements with carbapenem resistance in *A. baumannii* have focused on specific genes or were confined to small numbers of isolates (Poirel and Nordmann, 2006; Li et al., 2015; Vijayakumar et al., 2020). Our study on imipenem, a type of carbapenem antibiotic, resistance is broader and provides a whole-genome analysis of 349 isolates. The whole-genome approach we employed included an analysis of pairs of genomic factors; an important consideration for factors such as IS*Aba1* which have the potential to affect the expression of neighboring genes. Our approach, enabled by improved genome assemblies and genome scaffolding information, also allowed the assignment of factors to either chromosomes or plasmids, providing a better understanding of the potential for mobilization of the genomic factors identified.

Our analysis identified 10 genes that were strongly associated with imipenem resistance in the *A. baumannii* isolates we analyzed, and confirmed the association with resistance of genes identified by Wallace et al. when analyzing a subset of the genomes we explore here. Among the 10 genes we have found to be strongly associated with resistance, only *blaOXA-23* is widely believed to be involved in carbapenem resistance (Antunes et al., 2014); the other genes are associated with resistance to other antibiotics (Gutierrez et al., 2011; Alcock et al., 2020; Lin et al., 2020a), or are involved in the mobilization of resistance genes. This highlights a limitation of computational-only methods such as ours—causality can only be demonstrated experimentally. The results presented here provide several promising targets for experimental validation.

An intriguing finding of our study is that several genomic regions seem to be associated primarily with susceptibility to carbapenems even in the presence of known AMR genes. These genomic regions appear to contain a high density of transcriptional regulators, suggesting a potential role of these genes in the etiology of antimicrobial resistance in *A. baumannii*. Supporting the plasticity of these plasmids is the fact that a previously-sequenced variant of one of the plasmids we identified, *pORAB01-3*, contains an antimicrobial resistance gene that was not present in the genomes we analyzed. Given that the actual AST values of the chosen imipenem-resistant and susceptible isolates were close to the CLSI breakpoints used for determining the phenotypes, a thorough analysis of these genomic regions would be critical in understanding their roles in influencing antimicrobial susceptibility.

A second plasmid we have found to be associated with imipenem susceptibility, *pAYP-A2*, contains multiple genes that were statistically associated with imipenem susceptibility by Wallace et al. (2016), suggesting these genes may be in linkage disequilibrium, and highlighting a limitation of gene-centric analyses of antimicrobial resistance.

The study of AMR is critically dependent on collections of well-characterized isolates, and on the availability of high-quality genome assemblies for the isolates. Our analysis was enhanced by our use of scaffolding information generated from the paired-end sequencing data, as well as by our reconstruction of complete genome sequences for several isolates using a long read sequencing technology. Such resources enable the localization of genomic features associated with resistance within plasmids or specific chromosomal locations and allow for analyses of gene order/gene proximity that can reveal polygenic factors that impact resistance.

The strategy we have applied here does not rely on computationally-expensive multiple genome alignment approaches, rather focuses primarily on local similarities between genomes, and is, therefore, scalable and generalizable. We believe the approach we have used provides a template for similar analyses in other organisms, powered by the increasingly-available collections of isolates with well-characterized phenotypes (NCBI, 2021). Such datasets will provide fruitful sources of hypotheses for future experimental work, and expand our understanding of the genomic factors that underlie resistance to antimicrobials.

## 4. Materials and Methods

### 4.1. Primary Dataset

The *A. baumannii* isolates were obtained from a collection of patient surveillance samples collected as a part of a cohort study at the University of Maryland Medical Center (UMMC). These isolates were from patients treated in UMMC medical and surgical intensive care units. A total of 349 isolates were selected for evaluation. These isolates were cultured on selective media for *Acinetobacter* species and tested for susceptibility to imipenem using the Kirby-Bauer disk diffusion method. The phenotypes of these isolates were determined using the Clinical and Laboratory Standards Institute (CLSI) standard antimicrobial susceptibility testing guidelines: isolates with their susceptibility of at most 18 mm and at least 22 mm were considered imipenem-resistant and imipenem-susceptible respectively. The carbapenem susceptibility scores for these isolates, along with their NCBI identifiers, are provided in Supplementary Table 1. Additionally, a reference strain (Genbank ID: NC_017162) was used for the genomic analysis.

### 4.2. Genome Assembly, MLST Analysis, Gene Prediction, and Gene Clustering

All sequence read archive (SRA) runs for the 349 isolates were downloaded (449 SRA runs) and *de novo* assembled with SPAdes (v3.13.0) (default settings) (Bankevich et al., 2012). These genomes were further filtered to retain the high confidence contigs that had at least 10× sequence coverage. Accounting for the genome completeness in comparison with the reference genome, 426 assemblies were identified for the 349 *A. baumannii* isolates. Each of these 349 isolates was then represented by only one of its assembled genomes; the sequencing run corresponding to the representative assembled genome is the first of its associated runs mentioned in Supplementary Table 1. The quality of these selected 349 SPAdes assemblies as well as those available from NCBI were measured using QUAST (Gurevich et al., 2013)—the SPAdes assemblies were observed to be better in terms of number of contigs, misassembled contigs, unassembled contigs, variance between N50 and NG50 values, etc. (Supplementary Table 9), and were chosen for subsequent analyses.

*In silico* multilocus sequence type (MLST) profiles were assigned to the isolates using the sequences for the MLST markers from the Pasteur typing scheme (*gltA, recA, cpn60, fusA, pyrG, rpoB*, and *rplB*) (Diancourt et al., 2010). The genomes were scanned against the pubMLST database (http://pubmlst.org/abaumannii) using the mlst tool (Jolley and Maiden, 2010; Seemann, 2021).

Genes were then predicted from these assemblies using Prokka (v1.12) (Seemann, 2014). The gene annotations available for the *Acinetobacter calcoaceticus-Acinetobacter baumannii* (ACB) complex were downloaded from NCBI. These genes were clustered using CD-HIT (v4.8.1) with a length difference cut-off of 0.8 (-s 0.8) to create a custom protein database (Li and Godzik, 2006), which was then used for the gene prediction. The predicted genes were used to estimate the pan-genome for these isolates using Roary (v1.007002) with a minimum protein sequence similarity of 85% (-i 85) and gene paralogs were clustered together (-s) (Page et al., 2015). The pan-genome was also used for recognizing the assemblies that shared sufficient similarity for the downstream analysis. The known AMR genes were then identified by comparing the corresponding assemblies of these 349 isolates against CARD (Gutierrez et al., 2011; Alcock et al., 2020; Lin et al., 2020b).

### 4.3. Selection of Additional 13 Isolates for Chosen Microbial Clade Analysis

Among the isolates that did not host the 10 strongly correlated known AMR genes, two imipenem-susceptible isolates were genomically similar to 13 imipenem-resistant isolates within NCBI’s databases. One of these imipenem-susceptible isolates (Biosample: SAMN02384922) belonged to a SNP cluster (cluster-id: PDS000005681) on the NCBI Pathogen Detection Isolates browser (NCBI, 2021). Using this SNP cluster, 13 isolates were identified which had imipenem susceptibility information in the database. All 13 isolates were imipenem-susceptible and collected in clinical setting by the Multidrug-Resistant Organism Repository and Surveillance Network, Walter Reed Army Institute of Research (MRSN, WRAIR). The sequencing reads for these 13 isolates were downloaded and *de novo* assembled as before. Similarly, the genes were predicted from these genomes using Prokka. The pan-genome was estimated using Roary for the combined set of 28 isolates—13 imipenem-resistant and 15 imipenem-susceptible.

For further assessment, the 13 isolates were acquired from WRAIR. The following sections (4.3.1–4.3.3) describe the procedures undertaken on these 13 isolates for reculturing, phenotyping for carbapenem resistance, long read sequencing, and complete genome assembly.

#### 4.3.1. Biosafety, Media, Culture, and DNA Isolation

All work associated with the preparation and extraction of materials from the multi-drug resistant isolates were performed in a BSL-2 laboratory under the class II biosafety cabinet with appropriate PPE (disposable laboratory coat, gloves, N-95 respirators, and face shields) based on risk assessment. The *Acinetobacter baumannii* strains were grown using BD Bacto^*TM*^ Tryptic Soy Broth (Soybean-Casein Digest Medium). Single colonies were inoculated in TSB and grown overnight at 37°C. Cells were centrifuged and the pellet was washing using saline 0.85%. RNA-free genomic DNA was isolated using DNeasy Blood & Tissue Kits (Qiagen, Inc.), following the manufacturer’s instructions.

#### 4.3.2. Carbapenem Phenotype Testing

Antimicrobial susceptibility testing (AST) was performed for 12 of the 13 isolates obtained from WRAIR by broth microdilution and *E*-test (bioMérieux). For both broth microdilution and *E*-test (E-strips), we performed two culture passages for individual colony isolation on blood agar plates. A 0.5 McFarland suspension (10^8^ CFU/ml) was prepared to make broth microdilution plates cultures and a culture lawn on blood agar plates for E-strips. AST broth microdilution panels were incubated in aerobic incubation (36 degrees C) based on CLSI protocols. We utilized the Center for Veterinary Medicine’s AST meropenem and imipenem panels (CMV4AGNF and CMV2DW) respectively. The GN panel (CMV4AGNF) requires 4 QC isolates and the ESBL panel (CMV2DW) requires 5 QC isolates as quality control organisms. Vitek 2 Compact (V2C) (bioMérieux) was used for culture identification and BMD panels were read manually using the Vizion (Sensititre, Trek Diagnostics). Culture lawn on blood agar plates for E-strips were incubated upside down in aerobic incubation (36 degrees). Interpretive Criteria for categorizing susceptibility, breakpoints for both AST broth microdilution and E-strips were adopted from the CLSI document M100.

#### 4.3.3. Long Read Sequencing and Assembly

Six of the 13 *Acinetobacter baumannii* isolates, acquired from WRAIR, were sequenced on the Pacific Biosciences (PacBio) Sequel System (PacBio, Menlo Park, CA). Specifically, the DNA from the isolates were part of a 4-plex to construct multiplexed microbial SMRTbell library using the SMRTbell Template Prep Kit 1.0 (PacBio, Menlo Park, CA) according to the manufacturer’s protocol. The isolates were ligated with a unique barcode using the SMRTbell Barcoded Adapter Complete Prep Kit-96 (PacBio, Menlo Park, CA). Afterwards, size selection was performed with BluePippin (Sage Science, Beverly, MA). The multiplexed and size selected SMRTbell library was then sequenced on the PacBio Sequel sequencer using PacBio Sequel V2.0 chemistry on one Sequel SMRT cell 1M v2 (PacBio, Menlo Park, CA), with a 10h movie collection time. Raw sequencing data was demultiplexed by running the Demultiplex Barcodes application with the symmetric mode in SMRTLink v.7.0.1 (PacBio, Menlo Park, CA). The adapter sequence was trimmed and filtered out during the demultiplexing process. *De novo* assembly for the isolates was done using the PacBio hierarchical genome assembly process HGAP4.0 (Chin et al., 2013). The genomes of the 6 isolates were checked manually for even sequencing coverage, circularized by Circlator 1.5.5, and polished by “Resequencing” in SMRT Link v.7.0.1 to ensure > 99.99% mean consensus concordance (Hunt et al., 2015).

### 4.4. Comparisons of Specific AMR Associated Genes From Prior Findings

Similar to this study, a previous study by Wallace et al. had reported their findings based on genomic analysis of some other *A. baumannii* isolates collected as a part of the UMMC surveillance program (Wallace et al., 2016). Their analysis observed eight gene (centroid) sequences to be unique to carbapenem resistant isolates; these genes have not been previously considered to be AMR genes. The corresponding gene sequences were located in our assembled genomes using BLAST. The aggregated presence-absence counts for these eight genes is shown in Table 3.

Additionally, some other gene sequences were marked to be unique to these phenotypes within isolates grouped by their location of isolation—perirectal and sputum; these genes have also not been previously characterized as AMR genes. These comprised a total set of 385 gene sequences. We considered these genes to be strongly correlated with the resistance phenotypes, if they were reported to be unique to the corresponding resistance phenotype both overall or by source of isolation. The presence of these genes was determined across the available isolates using BLAST, and is shown along with the Fisher’s exact test statistics in Supplementary Tables 2, 6.

### 4.5. Genomic Structural Variants Detection and Lineage Estimation

The assembled contigs from SPAdes assemblies were scaffolded using MetaCarvel with -keep True parameter and -bsize 10 (without repeat resolution) to get the orientations of the contigs with at least 10 mate pairs linking these contigs (Ghurye et al., 2019). The relative orientations of the contigs, in conjunction with the coordinates of the genes, predicted using Prokka, facilitated the prediction of the potential presence of the insertion sequence IS*Aba1* (Accession:EF571004) in the upstream regions of various beta-lactamase genes.

The lineage of the isolates was estimated using Garli based on a single nucleotide polymorphism (SNP) matrix computed with the reference-based CFSAN SNP Pipeline (v2.1.0) using the reference genome (Genbank ID: NC_017162) (Zwickl, 2006; Davis et al., 2015). The CFSAN SNP Pipeline takes pair-end reads from each isolate along with the reference genome and provides a SNP distance matrix representing the pairwise proximity between the given isolates in terms of the SNP distance. This distance matrix was then used to hierarchically cluster the isolates with single linkage.

Structural variants within the aforementioned 28 similar genomes were detected *via* multiple whole genome alignment with Mauve (snapshot_2015-02-25 build 0) (Darling, 2004). Mauve constructs Locally Collinear Blocks (LCBs) to detect regions that are conserved across all or a subset of the given genomes. In addition, a custom script was used to detect the presence of shared *k*-mers (exact matching substring of length *k*) between these genomes and the genomic positions of these *k*-mers were used to locate the conserved contiguous regions. The figures for the gene cassette and the genomic locations of variants on the reference genome were made using the DNA Features Viewer library (Zulkower and Rosser, 2020).

The presence of genomic regions across the available isolates was ascertained using BLAST (blastn v2.8.1) or nucmer program from the MUMmer4 package (Altschul et al., 1990; Johnson et al., 2008; Marçais et al., 2018). The genomic region under consideration was deemed to be detected if the corresponding match resulted in the sequence identity of at least 90%.

### 4.6. Association With Resistance to Carbapenems

We combined the genes from the comprehensive antibiotic resistance database (CARD) and genes that have been reported to have associations with carbapenem resistance; this provided 2,650 genes that we take as the known antimicrobial resistance genes (Alcock et al., 2020). These known AMR genes were evaluated to check if their presence was statistically significantly correlated with the resistant isolates. We used Fisher’s exact test from the scipy.stats Python3 package to get the significance statistics for each gene (Virtanen et al., 2020). The entries of the 2×2 input matrix for the Fisher’s exact test corresponded to the number of isolates that were resistant and would have the predicted gene, those that are resistant and would not have the gene, and similar respective counts for the non-resistant isolates. A gene cluster was deemed to be significantly associated with resistance if the test resulted in a *p* ≤ 0.01. Since the number of resistant isolates largely exceeded the susceptible ones, we additionally constrained that a gene cluster was deemed significantly associated with resistant isolates if the test resulted in odds ratio ≥ 5 and was detected in ≤ 10 susceptible isolates. Among the 28 genetically similar isolates discussed before, owing to the high degree of genomic similarities, we constrained that a genomic variant (SNP or the conserved region) is strongly associated with a resistance phenotype if it is missing in at most one isolate of that phenotype (say resistant) and present in at most one isolate of the other phenotype (say susceptible).

## Data Availability Statement

The datasets presented in this study can be found in online repositories. The names of the repository/repositories and accession number(s) can be found in the article/Supplementary Material.

## Author Contributions

KJ performed the bioinformatic analyses, wrote the manuscript, and created the figures. NT and CP performed the microbiological and molecular lab work. MH and YL performed the PacBio-SMRT long read assemblies. SS performed the carbapenem phenotype testing. JKJ provided the carbapenem susceptibilities for the 349 *A. baumannii* isolates. JKJ, HR, PM, ES, and MP edited the manuscript. All authors read and approved the final version of the manuscript.

## Funding

This project was supported by the University of Maryland, Joint Institute for Food Safety and Applied Nutrition (JIFSAN) through cooperative agreement #5U01-FD001418, provided by the U.S. Food and Drug Administration, Center for Food Safety and Applied Nutrition (FDA, CFSAN). MP was supported in part by the NIH, award # R01-AI-100947.

## Conflict of Interest

The authors declare that the research was conducted in the absence of any commercial or financial relationships that could be construed as a potential conflict of interest.

## Publisher's Note

All claims expressed in this article are solely those of the authors and do not necessarily represent those of their affiliated organizations, or those of the publisher, the editors and the reviewers. Any product that may be evaluated in this article, or claim that may be made by its manufacturer, is not guaranteed or endorsed by the publisher.
