# Supplementary material for: Whole-Genome Assessment of Clinical Acinetobacter baumannii Isolates Uncovers Potentially Novel Factors Influencing Carbapenem Resistance
Source: Front Microbiol. 2021 Oct 1;12:714284. doi: 10.3389/fmicb.2021.714284 (PMC8518998; doi:10.3389/fmicb.2021.714284)
Supplement: Supplementary file 10 [file Presentation_1.PDF]

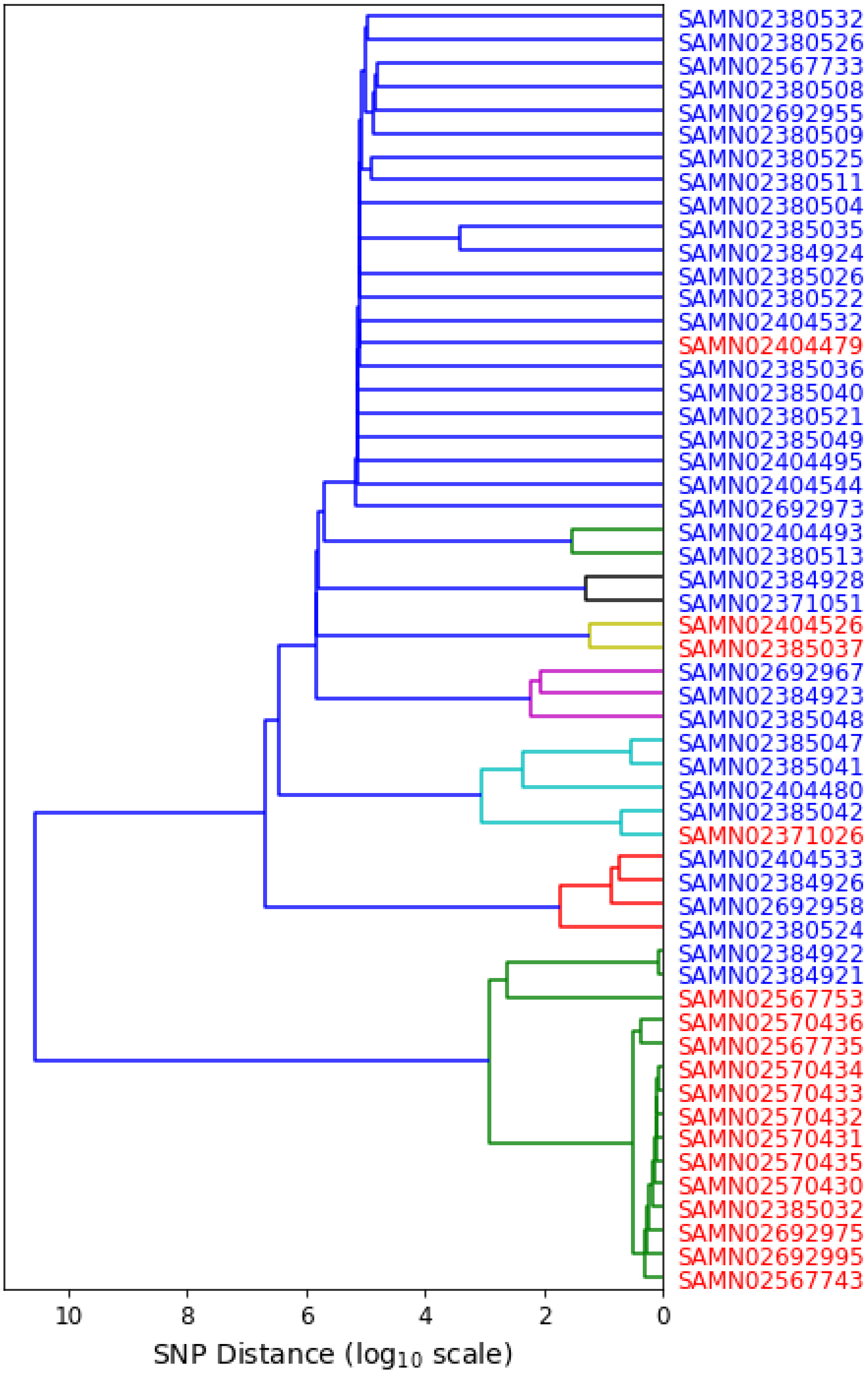

Figure S1: The estimated lineage of the 55 *A. baumannii* isolates that remain out of the initial 349 isolates which do not contain the ten known AMR genes whose predicted presence was strongly correlated with the imipenem-resistant phenotypes. The x-axis shows the isolates - those in red are resistant while those in blue are susceptible. The y-axis denotes the SNP distance obtained from CFSAN SNP Pipeline.
